# A Robust and Adaptable High Throughput Screening Method to Study Host-Microbiota Interactions in the Human Intestine

**DOI:** 10.1371/journal.pone.0105598

**Published:** 2014-08-20

**Authors:** Tomas de Wouters, Florence Ledue, Malgorzata Nepelska, Joël Doré, Hervé M. Blottière, Nicolas Lapaque

**Affiliations:** 1 INRA, UMR 1319 MICALIS, Domaine de Vilvert, Jouy-en-Josas, France; 2 AgroParisTech, UMR Micalis, Jouy-en-Josas, France; 3 INRA, US 1367 MetaGenoPoliS, Jouy-en-Josas, France; Rockefeller University, United States of America

## Abstract

The intestinal microbiota has many beneficial roles for its host. However, the precise mechanisms developed by the microbiota to influence the host intestinal cell responses are only partially known. The complexity of the ecosystem and our inability to culture most of these micro-organisms have led to the development of molecular approaches such as functional metagenomics, i.e. the heterologous expression of a metagenome in order to identify functions. This elegant strategy coupled to high throughput screening allowed to identify novel enzymes from different ecosystems where culture methods have not yet been adapted to isolate the candidate microorganisms. We have proposed to use this functional metagenomic approach in order to model the microbiota’s interaction with the host by combining this heterologous expression with intestinal reporter cell lines. The addition of the cellular component to this functional metagenomic approach introduced a second important source of variability resulting in a novel challenge for high throughput screening. First attempts of high throughput screening with various reporter cell-lines showed a high distribution of the response and consequent difficulties to reproduce the response, impairing an easy and clear identification of confirmed hits. In this study, we developed a robust and reproducible methodology to combine these two biological systems for high throughput application. We optimized experimental setups and completed them by appropriate statistical analysis tools allowing the use this innovative approach in a high throughput manner and on a broad range of reporter assays. We herewith present a methodology allowing a high throughput screening combining two biological systems. Therefore ideal conditions for homogeneity, sensitivity and reproducibility of both metagenomic clones as well as reporter cell lines have been identified and validated. We believe that this innovative method will allow the identification of new bioactive microbial molecules and, subsequently, will promote understanding of host-microbiota interactions.

## Introduction

Our body harbors an enormous amount of microorganisms of which 90% are found in the distal intestine. This highly dense ecosystem has repeatedly been shown to be tightly associated with its host, having a profound and sometimes unexpected impact on its health and well-being. If the equilibrated interplay between the intestinal microbiota, the host and ingested nutrients is disrupted, multiple metabolic, degenerative, inflammatory or infectious pathologies can emerge. Over the past years, the development of new animal models extended the understanding of the role of commensal bacteria to its implication in several physiological mechanisms from local effects on the epithelial barrier to systemic impacts on immunity and metabolism [1]–[3]. The intestinal microbiota contributes to intestinal homeostasis through direct regulation of the development of the intestinal mucosa and maturation of the immune system. Therefore the intestinal microbiota can be perceived as an integral component of the host’s physiology [3], [4].

The complexity of the ecosystem and the difficulty to culture most organisms has long prevented in depth functional exploration of this neglected organ. The development of molecular methods to explore this ecosystem summarized in the term metagenomics gave a new boost to gut microbiota research [5]–[7]. Originally based on cloning techniques, the advancement of sequencing technologies led to an increasingly profound genomic and genetic characterization of this diverse and variable ecosystem. Confirming what was known from previous, culture-based studies that the intestinal microbiota is composed of a very restricted number of phyla (mainly Bacteroidetes and Firmicutes) and that in contrast to this uniformity at high phylogenetic levels, there is a considerable inter-individual variability and thus phylogenetic diversity at a lower phylogenetic level [8].

This new sequence-based techniques to study the intestinal microbiota have led to numerous correlation-based studies exploring the interrelation of its composition with human health and various pathologies like inflammatory bowel diseases (IBD), obesity, diabetes and allergies [9], [10]. The increased genetic accessibility of the intestinal microbiota has further led to a more gene-centered understanding of the human microbiota, leading to a resurgence in interest for the huge genetic reservoir coded in the intestinal microbiome. In order to study the functional potential of the intestinal microbiota, a novel approach called functional metagenomics has been applied. This approach circumvents the challenging cultivation of individual intestinal bacteria by heterologously expressing the intestinal metagenome in a well-known, cultivable host (usually *E. coli*). Through this elegant approach, first studies have identified particular intestinal enzymes such as bile salt hydrolases fibrolytic enzymes, xenobiotic metabolizing enzymes like β-D-glucuronidases and prebiotic degradation pathways confirming the validity of functional metagenomics for intestinal bacteria [11]–[14]. Recent studies by our group have proposed to adapt functional metagenomics to study host-microbiota interaction *in vitro* [15], [16]. In our first studies, we demonstrated that the heterologous expression of the intestinal metagenome in *E. coli* is a valid tool to screen for bioactive compounds derived from the intestinal microbiota. Their activity patterns have been studied on different eukaryotic cellular models. First this combination was validated by studying the effect of 20725 metagenomic clones on intestinal epithelial cell line proliferation [16]. In a second step we established an intestinal epithelial reporters assay for NF-κB activation used to screen 2640 large fragment metagenomic clones for elements activating this nuclear receptor of crucial importance for intestinal inflammation responses [15]. The observed high variation and low hit rate of 0.8% when applying stringent criteria indicates that the screening of high numbers of metagenomic clones is necessary to identify bioactive compounds affecting host gene regulation. This can only be achieved in qualitatively exploitable way by a high degree of automatisation and adapted analysis techniques as used in high throughput screening for drug-discovery [17]. Unlike currently reported HTS assays, our screening workflow implicates two main independent biological systems: the metagenomic clones, and the cell-based assay. Combined they lead to a higher level of variability as commonly known from HTS. Cell-based HTS usually apply compound libraries to reporter systems [18], while HTS of metagenomic clone libraries have been tested for enzymatic activity on homogeneous defined substrates [11], [12]. Screening of metagenomic clones on cellular assays demands the control and eventual correction of two screens simultaneously. Amplified variability makes screening more challenging and thus requires robust and highly reproducible protocols and analysis tools.

In the present study, we describe a robust high throughput screening and analyzing method for the screening metagenomic clone libraries on reporter cell lines, adaptable to all the intestinal cell-lines tested. This method maximizes metagenomic clone expression, minimizes the edge effects and noisiness responsible for low confirmation rates observed in previous validation screenings. The capacity to screen large numbers (over 10000) of metagenomic clones on different intestinal cell lines enables the simultaneous screening of different intestinal metagenomes for a variety of mechanisms that genetically influence its host. Mechanisms underlying the complex host-microbiota interaction remain poorly understood and are of key importance to design microbiota related therapeutic targets. This innovative, robust and adaptable method will allow us to identify new bioactive molecular signals from the gut microbiota able to influence the host’s physiology and consequently the underlying mechanisms at the molecular level.

## Results

### Edge effects with former screening methods

**Figure 1:**
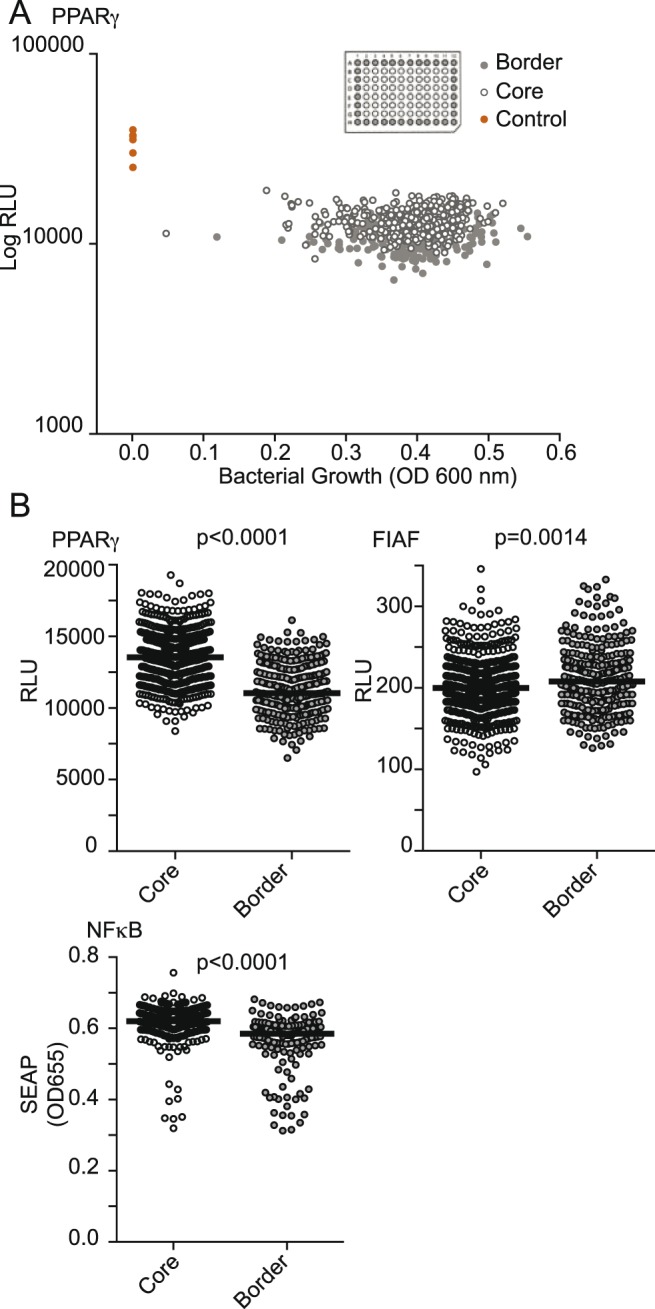
HTS using former protocol. A. Screening of 768 clones (8 plates) of a metagenomic library for PPARγ modulation using HT-29 reporter gene assay (RGA). Bacterial clone lysates were applied on HT-29-PPARγ (10% vol/vol) for 24 h and luciferase activity (RLU) was monitored. Border, core and control wells are represented in grey, white and red dots respectively. B. HT-29-PPARγ-luc, SW1116-ANGPTL4-luc and HT-29-NFkB-SEAP were seeded in 96 well-plates and homogeneously activated with sodium butyrate (2 mM, for PPARγ and ANGPTL4) or *E. coli* lysate (for HT-29-NFkB-SEAP) using a pipetting robotic workstation. 24 h after activation, luciferase (for PPARγ and ANGPTL4) and SEAP (for NFκB) activities were monitored. Border and core wells are represented in grey and white dots respectively. p-values indicated on the figure were obtained by a unpaired t-test of core *versus* border values.

We first applied the formerly reported screening protocol for HTS of metagenomic libraries [15]. Therefore, we developed several reporter gene assays (RGA) relevant for the study of host cell interactions with the microbiota, including one for ANGPTL4 and one for PPARγ activation. After completing the protocol described in the former experimental setup, we noticed a large spread of the response distribution in the first eight 96-well microtiter plates of the screening. The obtained data showed a position-dependent distribution of the signal over the plates on the used RGA (Figure 1A). The signal variation was specific to the RGA and conserved between plates (data not shown). Figure 1A shows the measures of the first screening on a PPARγ RGA. The data points plotted represent the growth of the applied bacteria (OD600) *versus* the reporter gene signal (luciferase activity measured in relative light units; RLU) as previously established [15]. Border and core wells were defined as described in Figure 1A and will be used for accordingly throughout the manuscript. Border and core wells of the screening form visibly distinct groups on the plot (Figure 1A) indicating a different range of responses for the relative parts of the plate. This effect is better visualized when border and core data points for all plates of the screening are separated and plotted as parallel dot plots side by side (Figure 1B). Statistical analysis confirmed the significance of this observation throughout the tested RGAs (Students t-test: p<0.001 for PPARγ and NF-κB RGAs, p = 0.0014 for the ANGPTL4 RGA). Following these observations, we analyzed and optimized the different steps of the screening protocol in order to establish a method robust and reproducible enough to be applicable for HTS of metagenomic libraries on different intestinal epithelial RGAs.

### Reduction of cell-related edge effects in RGA

**Figure 2:**
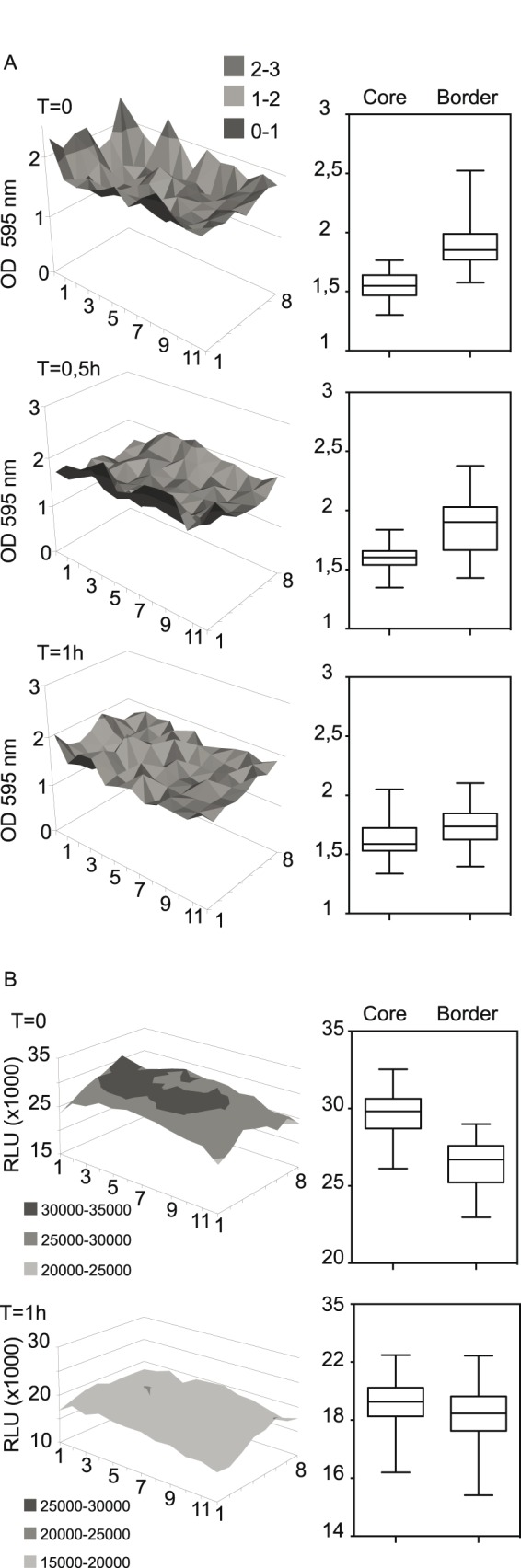
Edge effect reduction on cellular growth applied to the HT-29-PPARγ reporter cell-line. A Parental HT-29 cells were homogeneously seeded using a pipetting robotic workstation and pre-incubated at room temperature for the indicated time (0, 0.5 or 1 h) prior to 37°C incubation for 24 h. Cellular monolayer homogeneity was monitored using cell staining with crystal violet and quantified by absorption measurement at 595 nm. Left panel is a surface plot of a representative set of plates at increasing times of room temperature pre-incubation. Boxplot representation (right panels) of the OD 595 values for the border compared to the core of the respective representative plates. Mean p-values for different cell-lines tested are summarized in Table 1. B HT-29-PPARγ reporter cells were homogeneously seeded using a pipetting robotic workstation and pre-incubated at room temperature for the indicated time (0 or 1 hour) then at 37°C for 24 h prior to sodium butyrate (2 mM) activation. Luciferase activity (RLU) was quantified after 24 h of activation. Graph (left panels) shows two representative plates with different pre-incubation times at room temperature. Boxplots (right panels) represent the border and core values of the surface plots. Mean p-values for different cell-lines tested are summarized in Table 2.

Reduction of in-homogeneities in the cell monolayers was tested using methods previously described on adherent non-intestinal cell lines [19]. Therefore, the cell lines used for the construction of RGA assays (HT-29, SW1116 and Caco-2) were homogenously seeded and pre-incubated for 0, 0.5 and 1 h at room temperature prior to 24 h incubation at 37°C. In accordance to Lundholt *et al*., the in-homogeneities in the cellular monolayer were visibly reduced independently of the cell line as observed by light microscopy [19]. In the plates without any pre-incubation, the wells on the plate extremities showed cell agglomeration at the border and non-covered surface areas in their center leading to an in-homogenous cellular growth. This effect has been described to be caused by convection due to strong temperature gradients at the border positions of the plates when moved from room temperature to the 37°C incubator [20]. The effect could not be reduced by pre-warming the medium (data not shown). The homogeneity of cell growth and distribution was quantified using crystal violet staining after 24 h incubation at 37°C (Figure 2). Agglomeration of the cells at the border of the wells led to an increased retention of crystal violet in and between the cells and subsequently to a higher signal on the concerned position even though the cell did not cover the whole surface of the wells. The crystal violet staining technique therefore allowed quantification of the edge effect. As illustrated in Figure 2A (left panel), this in-homogenous cellular growth effect could be reduced by a pre-incubation time of 1 h at room temperature, allowing the homogeneous settling of the cells in the well before exposing them to the convection phenomena when introduced into the incubator. The right panel of Figure 2 shows the same data represented in box-plots separating core and border wells of the plate. The homogenization of the signal with increasing settling time at room temperature is clearly visible in both representations of Figure 2. Reduction of the edge effect was confirmed by statistical testing of the border *versus* the core wells performing a Student’s t-test on three independent repetitions (Table 1). The transferability of this edge effect reducing technique through homogenization of the cell sedimentation was confirmed on different reporter cell-lines (Table 1 and 2). Subsequently the responses of reporter systems were assessed in this setup. Figure 2B compares two representative PPARγ RGA plates homogenously activated with sodium butyrate with and without pre-incubation at room temperature using a surface plot. The reduction in edge effects can clearly be observed in both the surface plot and in the box-plot representation of the same data (Figure 2B). Reduction of the edge effect was also confirmed by statistical testing of the border *versus* core wells by a Student’s t-test on three different reporter cell-lines (Table 2). A pre-incubation of 1 h at room temperature allowing homogenous settling of the cells, is therefore a simple and efficient technique to reduce edge effects that can be applied to the used intestinal epithelial RGAs.

**Table 1:** Cellular growth edge effect reduction.

Mean p-values + SEM	T = 0	T = 1 h
SW1116	0.014+/−0.012	0.202±0.089
HT-29	0.023±0.023	0.063±0.062

Cell-lines were processes as in Figure 2 using crystal violet. Mean p-values +/− SEM comparing borders versus core for different parental cell-lines at 0 or 1 h room temperature incubation are reported.

**Table 2:** Cellular growth-dependent edge effect reduction applied to three different reporter cell-lines.

Mean p-values +/− SEM	T = 0	T = 1 h	Activator
HT-29 NF-κB-SEAP	0.002±0.002	0.248±0.147	*E. coli* lysate
SW1116 ANGPTL4-luc	0.000079±0.00008	0.202±0.068	Sodium Butyrate (2 mM)
HT-29 PPARγ-luc	0.023±0.015	0.239±0.108	Sodium Butyrate (2 mM)

**Figure 3:**
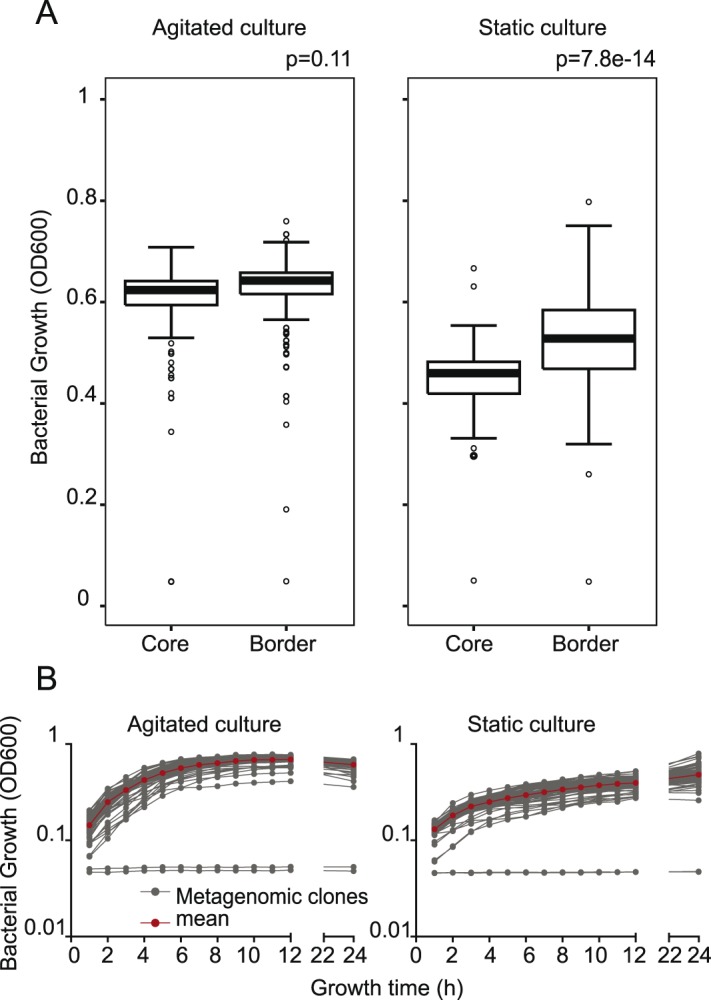
Metagenomic clones growth optimization. Four random plates of EPI300 metagenomic clones were inoculated from their −80°C glycerol stock into 96 well-plates (10% v/v) using a pipetting robotic workstation. After a 24 h pre-culture, they were re-inoculated and cultured overnight (10% v/v) at 37°C. These steps were performed either in static or agitated (700 rpm) culture conditions. Bacterial growth was monitored by optical density (OD 600 nm). A. Border and core wells of bacterial cultures in agitated (left panel) or static culture-conditions (right panel) for thus representative set of 4 plates. p-values indicated on the figure are the result of a unpaired t-test of the core *versus* border bacterial culture ODs. B. Growth curves of one representative plate of metagenomic clones (OD 600 nm) for agitated and static culture conditions. Red dots: mean OD600, joined grey dots: growth curve of single metagenomic clones.

Cell-lines were processes as in Figure 3. The table represents the mean of three p-values +/− SEM for comparing borders versus core for the different tested reporter cell-lines.

### Optimal bacterial growth conditions of metagenomic clones for HTS

Based on the large spread in bacterial growth in the first screening described above (Figure 1A), we decided to compare the formerly reported static growth conditions for the metagenomic clones with agitated growth conditions (Figure 3). Four representative 96-well plates with different metagenomic clones were cultured under both, agitated and static culture conditions. Pre-cultures were inoculated from the glycerol stock plates and cultured for 24 h at 37°C. The subsequent cultures were monitored by regular measurements of the optical density and are represented for one representative 96 well plate in figure 3B. The growth curves for each individual clone are represented by connected grey dots. The plates means for each time-point are represented as red dots. The growth curves of agitated culture show the canonical exponential growth phase followed by a stationary phase. We found that growth was more homogenous in agitated as compared to the static culture conditions, resulting in a narrower range of distribution of optical density (OD600) and consequently bacterial cell density in the plate (figure 3B). In addition, static growth conditions show no exponential growth phase, which might impact expression of the metagenomic clones insert (Figure 3B). Figure 3A shows box-plots of border and core data points for both agitated and static culture conditions. This representation further reveals that static growth conditions exhibit strong edge effects (p-value calculated by Student’s t-test is p = 7.8×10^−14^) while agitated culture shows no difference in growth distribution between core and border positions (p-value = 0.11). Agitated growth condition further highlight outliers due to impaired growth that are covered by the distribution under static growth conditions. These data clearly show that in order to achieve a better, homogenous bacterial growth associated with the elimination of edge effects, the use of agitated culture conditions is more appropriate for HTS since it guaranties the comparability of the single tested metagenomic clones.

Probability for heterologous expression can be increased and insert expression levels can be homogenized through optimization and homogenization of bacterial growth in all wells. Further most cell lines responses are biased, though in different extend by the growth level or the bacterial host of the metagenomic library. Therefore homogenization of metagenomic clones growth also means a homogenization of background noise.

### Homogenization of metagenomic insert expression and activity for HTS

**Figure 4:**
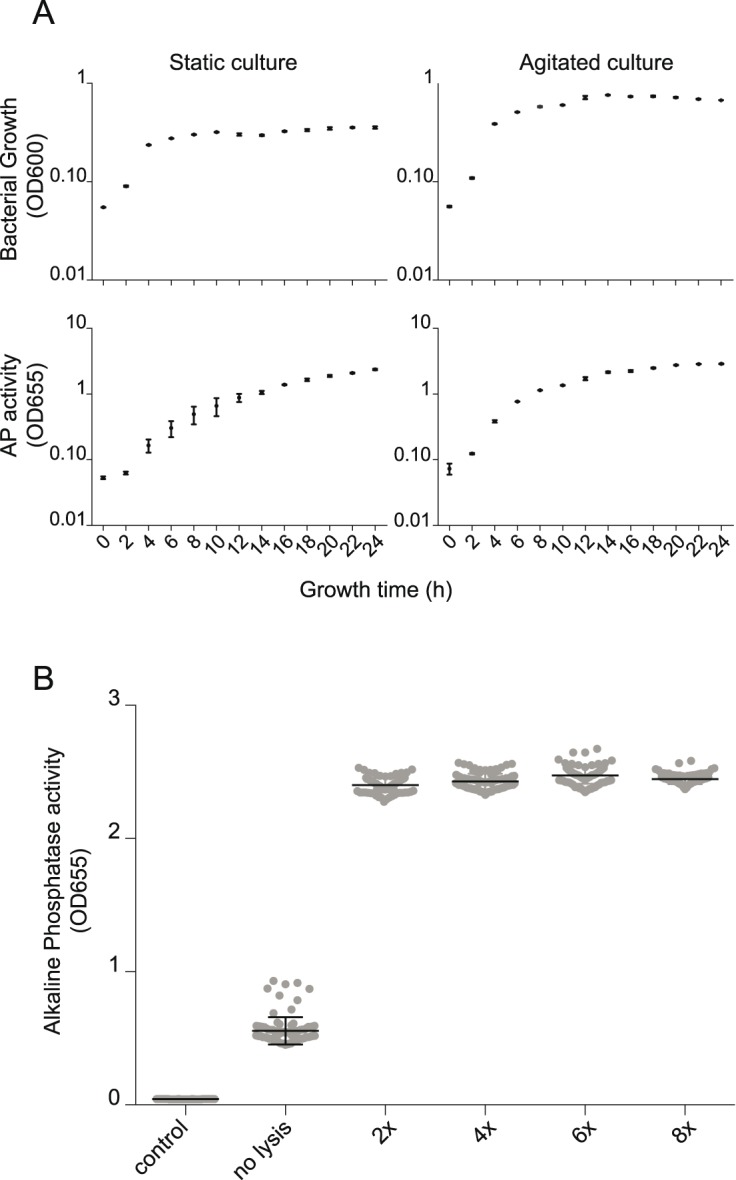
Bacterial growth, insert-dependent activation and lyses process optimization. The alkaline phosphatase-expressing clone 43C8 were cultured under agitated conditions, harvested at different times of growth, and subsequently lysed by congelation cycles (>1 h at 80°C). After filtration on 0.45 µm filters phosphatase alkaline substrate (QUANTI-Blue) was activated with bacterial lysates from different culture time-points. After 24 h, alkaline phosphatase activity was monitored (SEAP activity at OD655). A. represents the growth curve of the 43C8 (OD600) and the phosphatase alkaline activity of the respective lysates (AP activity at OD655). B. represents the alkaline phosphatase activity (OD655) under different lyses cycles (Control represents *E. coli* bearing an inactive fosmid with a human 40 kb-insert).

To screen metagenomic clone libraries for bioactive compounds we need to guaranty the expression of the insert and the availability of the bioactive compound. First can be done by defining the optimal expression conditions while for the second a lysis protocol, that does not impact bioactivity has to be established. In order to establish these protocols that guaranty homogenous growth, metagenomic insert expression and availability, we used a metagenomic clone named 43C8 exhibiting partially secreted alkaline phosphatase activity. Growth was monitored by the measurement of the OD600 while metagenomic insert expression was quantified using a colorimetric assay to monitor alkaline phosphatase activity (QUANTI-Blue reagent, OD655). The 43C8 metagenomic clone was grown in 96-well plates under agitated and static conditions. OD600 was monitored and samples were frozen at every time point prior to subsequent measure of the phosphatase activity. Data shown in figure 4A confirm suboptimal growth under static conditions as opposed to exponential growth under agitated growth conditions (700 RPM). For the metagenomic clone 43C8, it further confirms the hypothesis that optimal growth supports and homogenizes the expression of metagenomic inserts. Agitated culture shows exponential augmentation of phosphatase activity over time while static growth leads to a slower augmentation of activity and a higher spread within the four independent wells used for the experiment. Figure 4A further suggests that metagenomic insert expression can be delayed under unfavorable growth conditions. We therefore suggest a culture time of at least 16 h covering all bacterial growth phases to reach full expression of the metagenomic insert circumventing potential growth phase dependent expression.

Metagenomic clones used for screening carry a 40 Kb insert of heterologous DNA. Even though whole operons have been described in metagenomic clones, the secretion of eventual activating compounds cannot be presumed. We therefore decided to lyse the metagenomic clones in order to solubilize intracellular compounds.

In order to conserve integrity of eventual bioactive compounds lyses using freeze thaw cycles was chosen. As represented in the parallel dot plot of entire 96-well microtiter plates in Figure 4B, lysis not only homogenizes the activity of the single clones within the plate but also renders the intracellular compounds accessible thereby increasing the signal of the 43C8 clone. Even though the repetition of freeze/thaw cycles further homogenizes metagenomic clone activity, two freeze thaw cycles seem to be the most adequate lysis for HTS application showing a robust result with manageable effort.

### Bacterial and cellular growth optimal protocols reduce the background noise and increase the reporter assay sensitivity

**Figure 5:**
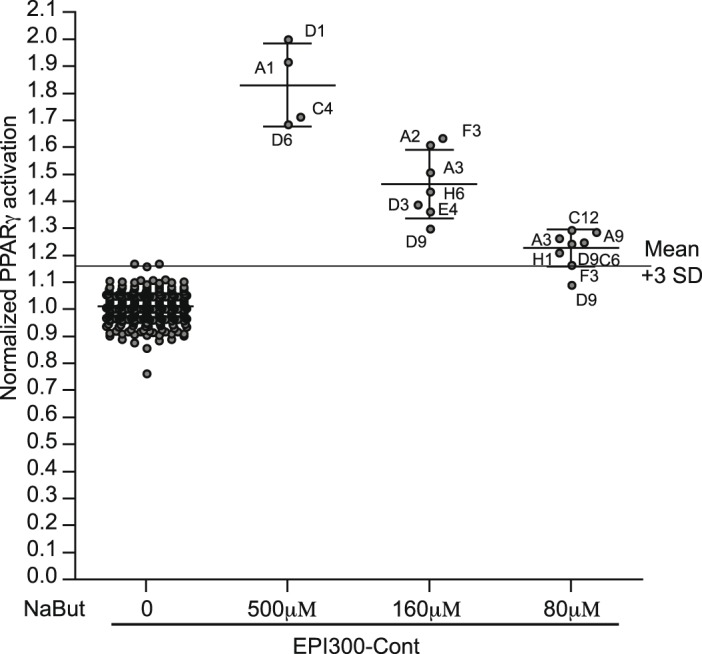
Detection limit on optimized reporter gene assay. The HT-29-PPARγ cell-line was homogeneously seeded using a pipetting robotic workstation and incubated at room temperature for 1 h prior to 37°C incubation. After 24 h of culture, all wells were activated homogenously with EPI300-Cont lysates for 24 h at 37°C. In randomly chosen wells (50% border and 50% core), three different concentrations of PPARγ activating NaBut (at 500 µM, 160 µM and the sub-activating concentration 80 µM) were randomly added in the 96 well-plates. The graph represents normalized values from 3 independent plates.

The newly established protocols were combined and tested for their effect on reporter assay sensitivity. Intestinal epithelial cell lines display differential amounts of activation or modification upon application of lysates of *E. coli* such as used for the functional metagenomic screening. RGA used to screen host-microbiota interactions, such as NF-κB, Angptl4 and PPARγ are known to be activated by bacterial compounds other than coming from metagenomic insert. Therefore the background noise for the screening is a combination of RGA noise and bacterial growth homogeneity. Thus we tested the sensitivity of screen when applying the previously established modifications. We randomly applied three serial dilutions of the PPARγ activating component sodium butyrate (NaBut; 500 µM, 160 µM and the sub-activating concentration 80 µM) equally to border and core positions of a RGA plate using the previously mentioned optimization techniques. The HT-29 PPARγ reporter cell line was seeded and pre-incubated for 1 h at room temperature before 24 h incubation at 37°C. Prior to NaBut stimulation, all wells were homogenously activated with a filtered lysate of the *E. coli* strain culture used for the metagenomic library, namely EPI300-Cont, to simulate screening conditions. Figure 5 represents the PPARγ response to the three NaBut concentrations from three independent plates. The position of the individually activated wells is indicated next to the plotted points. Wells with activator concentrations of 500 µM (EC50, Figure S1) and 160 µM NaBut were clearly identifiable. Interestingly, even with a high background signal related to the control *E. coli* EPI300-Cont stimulation, we were able to identify activation with a concentration of NaBut at its detection limit (80 µM) for most of the wells. Assuming a normal distribution of data-points the threshold for hit detection is set at 3 standard deviations from the mean (mean+3 SD). A normalization versus the mean as illustrated in Figure 5 allows the detection of clear signals. The cutoff of 3 standard deviations however generates falls positives and negatives even at this small scale and with known activators. We therefore tested methods to minimize position effects and clearly eliminate false positives and negatives in this controlled setup.

### Normalization as an additional tool to increase the robustness of functional metagenomic HTS

**Figure 6:**
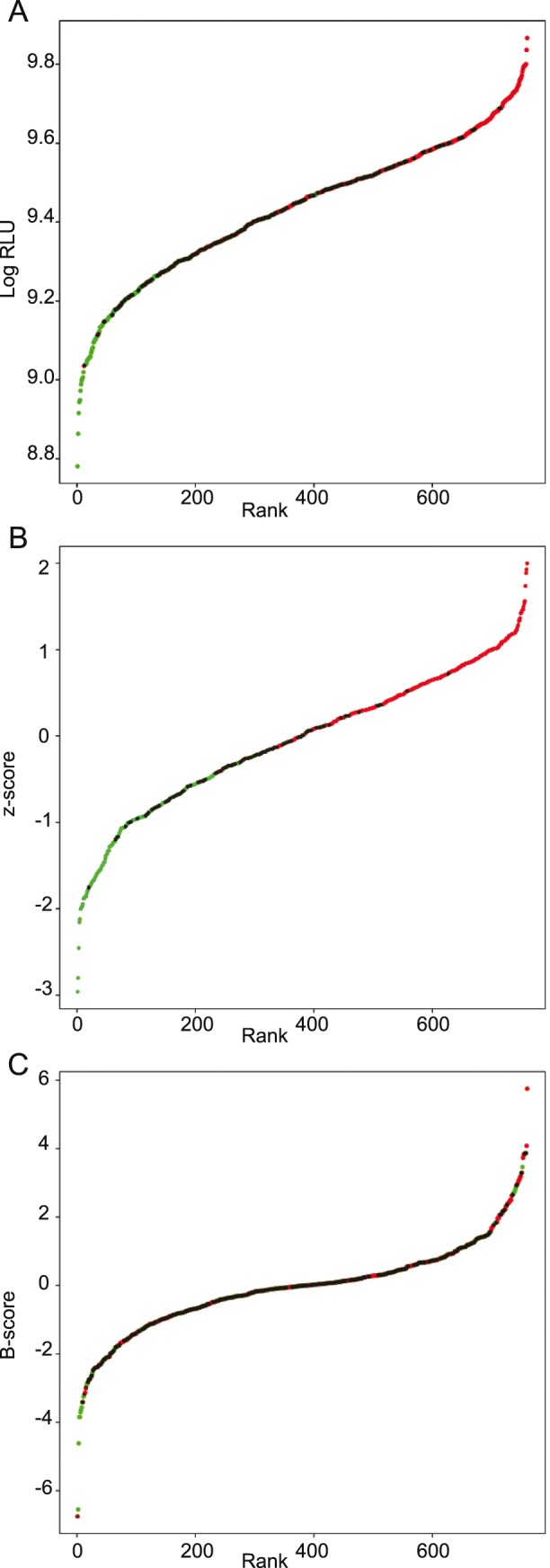
Different normalizations of assay plates. Different mathematical approaches for normalization were applied to the dataset from the initial HT-29-PPARγ screening. A shows all data-points as dots ordered by activity-rank whereby the red dots are points on the plate’s cores and the green points are well on the borders. Dark green areas show overlapping of red and green dots. B shows ranked Z-score normalization of the results. (red dots are points on the cores and the green points are well on the borders. Dark green areas show overlapping of red and green dots. C. shows the ranked B-score values for the same data set. (red dots are points on the cores and the green points are well on the borders. Dark green areas show overlapping of red and green dots.

**Figure 7:**
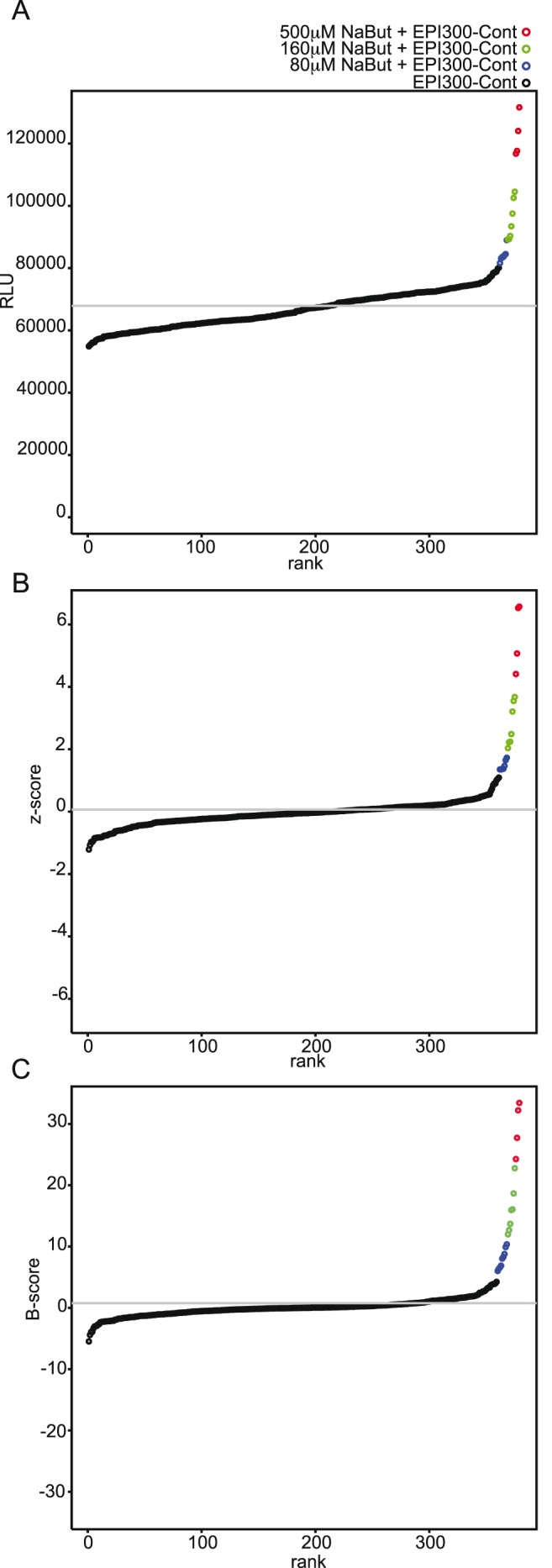
Detection limit of the screening protocol. Statistical normalization applied to optimized cellular and bacterial culture conditions. As in Figure 5, HT-29-PPARγ cell-line was homogeneously seeded using a pipetting robotic workstation and incubated at room temperature for 1 h prior to incubation. After 24 h homogenous EPI300-Cont lysates were applied to the cell assay (1/10 v/v) for 24 h at 37°c. In randomly chosen wells (50% border and 50% core), three different concentrations of PPARγ activating NaBut (at 500 µM, 160 µM and the sub-activating concentration 80 µM) were randomly added in the 96 well-plates. The resulting measured luciferase activity (expressed in relative light units, RLU) is represented as activity-ranked dots. Luciferase activation for EPI300-Cont, EPI300-Cont+500 µM NaBut, EPI300-Cont+160 µM NaBut and EPI300-Cont+80 µM NaBut are represented in black, red, green and blue respectively. Grey bars represent mean activation (Mean) and mean activation +/− standard deviation (SD).

In order to increase robustness and sensitivity of our assay, different data treatment strategies were tested. Since protocol optimization has reduced edge effects dramatically, but does not eliminate position effects completely in all the reporter cell-lines used (Figures 1–5 and data not shown), we tested different data analysis protocols for their capacity to reduce positional effects found in all biological screenings and so increase the sensitivity and robustness of our assays. After homogenization of metagenomic clone culture conditions and thus elimination of expression bias due to growth, our readout can be considered to be one dimensional, as opposed to the two dimensional view defining each data point with the RGA signal and the metagenomic clones culture OD600. We consequently decided to present the data as ranked spots in order to better visualize cut-offs at both extremes (Figure 6). Raw data from the reported first screening on HT-29-PPARγ were taken for analysis (Figure 1A). Figure 6A shows the ranked untreated data where red dots represent data points from the core wells and green points represent data points from the border wells of the 96-well plates. Superposition of green and red points results in dark green points, indicating the part of the curve where border and core data point overlap. The separation of core and border data points showed previously in Figure 1A is also clearly visible in this representation (Figure 6A) where both extremes are dominated by one color. The frequently used normalization method Z-score facilitates inter plate comparison, but does not eliminate position effects of the plates as the clear separation of border and core in Figure 6B illustrates. To consider the observed position effects we used a more appropriate normalization method less susceptible to outliers and position effects namely the B-score. Roughly in order to take into account column and row effects the B-score represents the ratio of an position adjusted raw value to a measure of variability in the plate named Median absolute deviation (MAD) [18], [21], [22]. The resulting normalized data are shown in Figure 6C. B-score normalization reduces the positional effects and simultaneously accentuates the tails of the distribution indicating that B-score normalization is an appropriate tool to address the position effects in our system. We then applied the B-score normalization to the dataset represented in Figure 5 where randomly-picked wells of the core and border of the plates were stimulated with RGA activating NaBut down to its previously defined activation limit (Figure S1). This dataset generated using the optimized protocol had a lower spread of its data point distribution (Figure 7). The reduction of variation visibly improved the detection of activators. Ranked raw data shown in Figure 7A already allows the detection of strong activators quite robustly at a small scale, tough low activators are partially covered by the variation of the background noise. Z-score normalization allows an inter-plate normalization but does not increase sensitivity of the assay since the Z-score is sensitive to outliers; in this case, the activated wells (figure 7B). The activated wells increase variability and thus the recommended cut-off of mean±3 SD used to define hits covers a large part of activators. In contrast, B-score normalization is insensitive to position effects. Applied to the same dataset, Figure 7C shows that B-score normalization cannot only smoothen the distribution through its elimination of position effects. It also accentuates the outliers, and allows the low concentration of activators to be clearly identified through a clear-cut separation from the background noise. For our screening setup, B-score normalization, therefore, avoids the loss of hits as false negatives due to background noise and simplifies the identification of hits through its accentuation of outliers. The combination of an experimental reduction in variation completed with statistical elimination of position effects resulted therefore in a highly sensitive assay, meeting all requirements for a High Throughput Screening assays on host-microbiota interactions.

### Screening using the normalization protocol for identification of metagenomic clones responsible for bioactive effects

**Figure 8:**
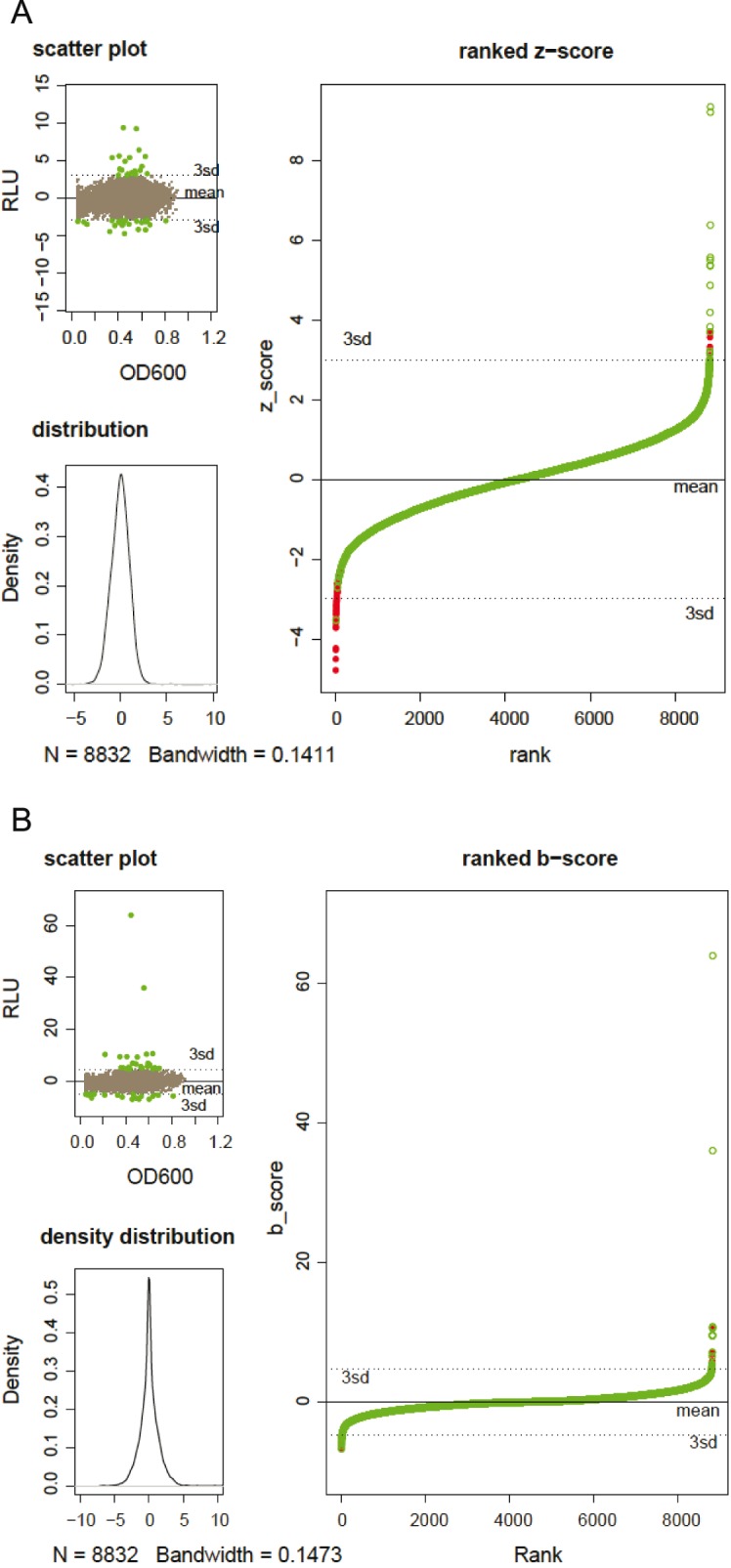
PPARγ HTS screening. Comparison of different data analysis methods of screening data from the screening of 92×96 metagenomic clones on a PPARγ RGA. Figure A represents Z-score normalized data B represents B-score normalized data. The top left picture plots the each readout in function of the growth of the metagenomic clone. The bottom left picture represents the distribution of the readouts over the whole dataset and the large picture on the right part represents all data points ranked by readout. The coloration of red dots for the borders and empty green dots for the core allow to visualize eventual position effects.

The established protocol was used to screen 92 microtiter-plates with a total of 8832 clones (Figure 8). Analysis of Z-score normalized and B-score normalized data confirms our previous observations that, even with an optimized protocol reducing position effects in all steps of the screen, there remains a position bias. Figure 8A where data points on the border are plotted in green and wells of the core are plotted in red illustrates the position bias Z-score normalization propagates. This makes it impossible to identify clones using the common criteria of intra-plate Z-score normalization.

B-score in contrast showed a much narrower distribution where inactivated wells are concentrated to the mean (Figure 8B). This distribution with its accentuation of outliers allows a clear separation of background-noise and positives on the ranked plot and thus a better identification of hits. Therefore the established method combined with B-score normalization confirms to be suitable for HTS of metagenomic libraries on RGAs.

The strategy for functional metagenomic assays aims to the identification of the bacterial genes involved in the metagenomic clone’s activity on the selected reporter assay. Once hits are identified in the screening downstream processing of confirmation screening and identification of bioactive compounds will be performed in a small scale. The presented method is thus a powerful tool to identify bioactive compounds of the intestinal microbiota. Downstream processing however has to be adapted to the nature of the identified activator and is thus not subject of this study [15].

## Discussion

An increasing number of studies emphasize the diverse roles of the intestinal microbiota. These roles are often associated with beneficial physiological effects for the host, reflecting a symbiotic and beneficial crosstalk between cells of the intestinal epithelium and the resident microbiota. However, the cellular and molecular mechanisms developed by the microbiota in order to influence the host’s intestinal cell responses remain poorly understood.

An emerging and innovative field called functional metagenomics, has led to the discovery of a variety of novel genes and their associated functions. This approach was first applied to the intestinal microbiota to identify enzymes such as bile salt hydrolases, fibrolytic and xenobiotic metabolizing enzymes [11]–[13]. This method has proven to be effective since the well-known, cultivable *E. coli*, used as host, is able to express up to 40% of the functional potential from randomly cloned environmental DNA. Among others, new enzymes from anaerobic and Gram-positive bacteria have been identified by heterologous expression of candidate metagenomes in the easy cultivable *E. coli* [23]. We previously proposed the use of a functional metagenomic approach to model the host-microbiota interaction *in vitro* [13], [15]. Importantly, previous functional studies showed that hit rates in metagenomic screenings are expected to be in the range of less than 0.01%, therefore requiring high throughput screening in order to identify molecules of interest. Previous metagenomic high throughput screenings (HTS) were performed to identify enzymatic activities on well-defined substrates. Contrary to these first metagenomic screenings, the study of host cell-microbiota interactions described here has to take into account two independent biological systems: the metagenomic clones, and the cell-based assay, representing two independent sources of variability and noise for the screen. This was observed by using the former experimental setup on different reporter cell-lines (Figure 1 and data not shown) where we noticed a position-dependent and highly spread distribution of the response. These sources of variations and consequent difficulties to reproduce the observations are not compatible with HTS since they impair the ability to robustly identify hits, leading to high numbers of false positive and negatives. We therefore developed a robust and highly reproducible protocol and defined appropriate analysis tools in order to be able to exploit this promising new approach on a broad range of reporter-cell line assays.

**Figure 9:**
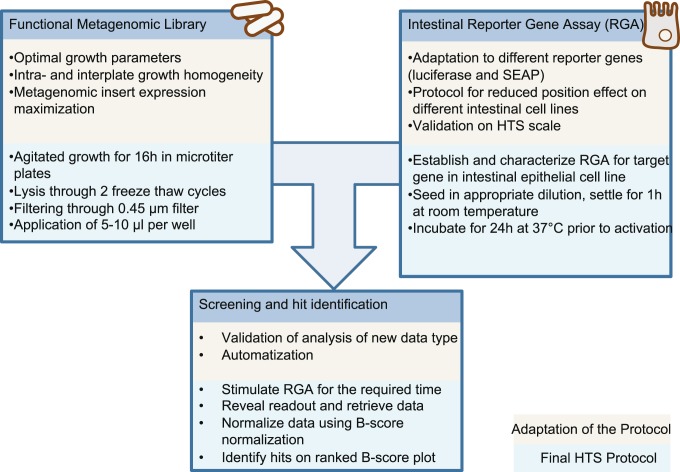
Schematic overviewed of challenges addressed and the resulting suggested protocol.

In the present study, we established the conditions necessary to perform a robust, reproducible high throughput screening assay combining metagenomic clones and cellular models. This allows us to identify bioactive motives from the human intestinal microbiota on different intestinal cell-line models, and hence screen the intestinal microbiome for the single and eventually rare mechanisms implicated in host-microbiota cross-talk. In this optimal setup, we minimized the edge effects, variation and noisiness of the assay, we had previously identified as the source for high insecurity level for hit definition and subsequently low confirmation rates. The optimal methods implies the use of a robotic pipetting workstation, a 16 h agitated bacterial culture and a 1 h room temperature settling time for the reporter cell-line. The increased control of the screening process is completed by appropriate data treatment, namely the B-score normalization, which showed to be an appropriate method to normalize increased the robustness and sensitivity to a level compatible with HTS. These adaptations provided clear identification of minimal activator concentrations with a threshold of as little as one standard deviation, which demonstrates that HTS can be performed on combinations of two biological systems like the combination of metagenomic clones with cell models to identify interactions. We believe that the presented approach as summarized in Figure 9 can be used to screen different gene pools reaching from complex ecosystems microbiomes to genomic approaches for single microbes.

The understanding of the complex host-microbiota relationship and the possibility to modulate these interactions is of key importance for human health. Our knowledge on the various contributions of the microbiota to health is still in its infancy and the underlying cellular and molecular mechanisms of its interplay with the host intestinal cells remain poorly understood. We believe that this innovative and adaptable method will allow the identification of new bioactive molecules from the intestinal microbiota able to influence the host’s physiology on various levels such as immunity, metabolism or tissue differentiation. Using this optimized method, we have already identified over 30 clones of interest which are being fully characterized using the additional experimental steps previously described [15]. Expanding this new form of HTS to new targets and ecosystems is a challenge that will build on this first experience. Identifications of the bacterial actors of such interplay will lead to the molecular characterization of the mechanisms underlying the microbiota’s properties. This approach will allow the identification of molecular signals from yet totally undescribed micro-organisms as well as novel mechanisms of the host responses. Considering the importance of host-microbiota interactions for human health, the access to yet unexplored resources will lead to the discovery of new actors regulating this cross-talk and therefore new potential therapeutic targets.

## Materials and Methods

### Cell Culture and Reagents

HT-29 (ATCC HTB-38) and SW1116 (ATCC CCL233) cells were from ATCC and maintained in RPMI 1640 medium in a 5% CO2 atmosphere at 37°C. Both media were complemented with 10% fetal calf serum, 10 mM sodium pyruvate, 10 mM nonessential amino acids, 2 mM glutamine, penicillin (50 U/ml), and streptomycin (50 U/ml) (all from Lonza).

### Plasmid Constructs

The ANGPTL4 promoter sequence was amplified from human genomic DNA using the HiFi DNA polymerase (Fermentas) according to the manufacturer’s instructions. Briefly, primers used for ANGPTL4 PCR-amplification were fw:TTTTTTGCTAGCCTCAGGACATTAAAGACCCTGGCGGTAGAG and rw: TTTTTTGGTACCCCTCTTAGGTAGCCTGGGAGCGGGGATTCG. The ANGPTL4 promoter construct (consisting of the 1.8 Kb upstream transcriptional start) was cloned into a modified pcDNA3.1/Zeo (harboring luciferase and without any CMV promoter sequence, kind gift from A. Cultrone, INRA, France) and verified for authenticity by sequencing [24]. PPARγ reporter construct pJ3-TK-Luc was from M. Chamaillard, (INSERM Lille, France) pTK-Hygro was from Invivogen.

### Transfection and stable cell-lines selection

Transfections were performed using TFX50 (Promega), according to manufacturer's recommendations. Stable reporter cell lines for PPARγ were cotransfected with pTK-Hygro (Clontech) and the ANGPTL4 promoter sequence, were selected by selective pressure of the appropriate antibiotic (600 µg/ml Hygromicin, 50 µg/ml Zeocin). The NF-κB reporter clone HT-29/kb-seap-25 has been described previously [15].

### Reporter cell-lines activation and analysis

All experiments were performed using a robotic pipetting workstation (Hamilton Robotics Star Line) for high seeding, stimulation and revelation homogeneity. For each experiment, cells were seeded at 25000 cells/well in 96 well-plates and incubated 24 h prior to activation. Homogeneous cellular activations were performed using sodium butyrate (NaBut, 2 mM, Sigma) for PPARγ and ANGPTL4 reporter systems or 0,45 µm-filtered, sonicated *E. coli* (Epi300 from Epicentre, 10% vol/vol) for NFκB-SEAP system. Cells were then incubated 24 h prior quantification of the reporter gene expression (alkaline phosphatase (SEAP) or luciferase). SEAP (for NFκB system) secretion or intracellular luciferase expression (for both ANGPTL4 and PPARγ systems) were revealed using the QUANTI-Blue (Invivogen) or One-Glo (Promega) reagent respectively, according to the manufacturers’ instructions. All measurements were performed using a microplate reader (Infinite 200, Tecan) and quantified at 655 nm OD for SEAP and by luminescence for luciferase.

### Crystal violet staining

Cellular growth was monitored using crystal violet absorption property at OD 595 nm as previously published [25]. Briefly, HT-29 or SW1116 cell-lines were homogeneously seeded using a pipetting robotic workstation and incubated at room temperature for the indicated time (0–1 h) prior to 37°C incubation for 24 h. Then, cells were fixed in methanol at −20°C for 15 min prior incubation with 0.1% of crystal violet in deionized water for 30 min. Subsequently, cells were extensively washed in water. Crystal violet retained in the cell layer was solubilized in 100 µl 0.2% Triton X-100 (Sigma) and quantified through absorption measurement at 595 nm using a microplate reader (Infinite 200, Tecan).

### Metagenomic clones

Randomly selected plates from the metagenomic library issued from the intestinal microbiota of obese and lean patients (constructed using CopyControl Fosmid Library Production Kit by Genoscope, France) were used in this study. The library consists of *E. coli* EPI300T1R clones carrying each one fosmid with a metagenomic inserts of approximately 40 kb. *E. coli* bearing an inactive fosmid with a human 40 kb-insert (ligation control reaction for CopyControl Fosmid Library Production Kit), referred to as EPI300-Cont, was used as control. Bacteria were grown under static or agitated conditions in 96 well-plates in LB broth (Lennox L broth base, Invitrogen) at 37°C. Bacterial growth was monitored by absorbance measurement at 600 nm, using a microplate reader (Infinite 200, Tecan). We used a metagenomic cloneshowing partially-secreted alkaline phosphatase activity, namely 43C8, as a read-out for bacterial lysate activity. Bacterial lysates were obtained through freezing at −80°C and subsequent thawing at room temperature. Bacterial lysates were filtered by centrifugation through a 0.45 µm microplate filter (Corning) prior to use on reporter cell-lines.

### Statistical analysis

Graphic representations and statistical analysis were performed using the Graphpad Prism (GraphPad Software, San Diego California USA) or R statistics softwares (www.r-project.org/). Comparisons of distributions were performed using an unpaired t-test with 95% confidence intervals. p Values smaller than 0.05 (p≤0.05) were considered significant. Z-scores and B-scores were calculated as described by Malo et al. using R statistics software [18]. Median polish was performed plate-wise based on the Tukeys method [26] using the R command medpolish(x). Residuals were used for further calculation of the B-score (medpolish(x)$residuals).

## Supporting Information

Figure S1 **Dose response curve of sodium butyrate on the PPARγ RGA used in this study.**
(EPS)
